# Type 2 immunity to the rescue: enhancing antitumor immunity for skin cancer prevention

**DOI:** 10.1172/JCI188018

**Published:** 2025-01-02

**Authors:** Matthew D. Vesely, Sean R. Christensen

**Affiliations:** 1 Department of Dermatology, and; 2 Department of Surgery, Division of Plastic and Reconstructive Surgery, Yale School of Medicine, New Haven, Connecticut, USA.

## Abstract

Cutaneous squamous cell carcinoma (cSCC) incidence and deaths continue to rise, underscoring the need for improved cSCC prevention. Elimination of actinic keratosis (AK) precursor lesions is a major strategy to prevent cSCC. Topical calcipotriol and 5-fluorouracil (5-FU) have been shown to eliminate AKs and reduce the risk of cSCC development, but the mechanism was undefined. In this issue of the *JCI*, Oka et al. demonstrate that type 2 immunity is necessary and sufficient for the elimination of premalignant keratinocytes and cSCC prevention. Paired biopsies from AK lesions and unaffected skin revealed that only keratinocytes from AKs produced thymic stromal lymphopoietin (TSLP) and damage-associated molecular patterns, resulting in selective recruitment of Th2 cells to the AK lesion. In mouse models of skin carcinogenesis, TSLP was necessary to recruit Th2 cells and trigger IL-24–mediated keratinocyte cell death. These findings suggest that the TSLP/Th2/IL-24 axis is a potential therapeutic target for SCC prevention.

## Squamous cell carcinoma and field cancerization

Cutaneous squamous cell carcinoma (cSCC) is the second most common cancer in humans, accounting for over 1,000,000 cases and 5,000 deaths per year in the United States, and the incidence continues to rise (1–3). The primary risk factor for development of cSCC is exposure of the skin to mutagenic ultraviolet radiation (UVR) from the sun (1). Unlike many internal malignancies, cSCC frequently presents with multiple primary tumors. Among patients with an initial diagnosis of cSCC, 42% will develop additional new primary cSCC within 5 years, and this risk increases to 72% in patients with two or more cSCCs (4). A small fraction of patients, particularly those with immunosuppression, develop more than 10 primary cSCCs, and these patients exhibit markedly increased risk for metastasis and death (5).

Detection, treatment, and prevention of multiple primary lesions is a cornerstone of management of cSCC. The existence of multiple tumors within a region of skin subjected to UVR is termed field cancerization and is a unique feature of cancers of the skin (6). While the development of systemic immune checkpoint inhibition has revolutionized the treatment of locally advanced or metastatic cSCC (7), these treatments do not necessarily solve the challenge of subsequent primary cSCC in patients with field cancerization (8). More effective and durable prevention strategies for cSCC are needed. In this issue of the *JCI*, Oka et al. describe the immunologic mechanism that may underlie a promising approach to cSCC prevention (9).

Patients with cSCC typically present with numerous precursor lesions of actinic keratosis (AK) prior to and simultaneously with development of cSCC (10). Although AKs are premalignant lesions that are not dermally invasive, they share many of the same UVR-induced driver mutations as cSCC, including loss-of-function mutations in *TP53* and *NOTCH1* (11). Treatment of premalignant AKs represents an attractive strategy for cSCC prevention. Several treatments are available for the elimination of premalignant AKs, including topical 5-fluorouracil (5-FU), topical imiquimod, topical tirbanibulin, and photodynamic therapy, but at present only topical 5-FU chemotherapy has been shown to reduce the incidence of subsequent cSCC (12). Moreover, none of these treatments for AKs have been demonstrated to induce durable immunity to cSCC or its precursors. Work from the Demehri laboratory has previously shown that topical 5-FU in combination with calcipotriol (a topical vitamin D analog used to treat psoriasis) can effectively eliminate AKs after a four-day treatment regimen in an exploratory clinical trial in humans (13). Furthermore, combination 5-FU/calcipotriol treatment reduced the incidence of subsequent cSCC development over a 3-year period (14). While the authors demonstrated that this response was associated with infiltration of skin lesions with CD4^+^ T cells, the immunologic mechanism remained undefined (14).

## Type 2 immunity in cancer immunosurveillance

In the current manuscript, Oka et al. first performed an open-label trial of 5-FU plus calcipotriol twice daily for 6 days in participants with pre- and postbiopsies of AK and adjacent unaffected skin to assess the immune response (9). These experiments confirmed a marked reduction in clinical AK after 5-FU-plus–calcipotriol treatment and identified infiltration of treated AKs with T helper 2 (Th2) cells expressing CD4 and GATA3. Other T cell subsets (Th1, Th17, and regulatory T cells) were not increased in treated AKs, and treated normal skin did not show infiltration of Th2 cells. Interestingly, Oka and authors also performed follow up studies of patients from their original trial after 5–7 years. Residual AKs showed infiltration with GATA3^+^ Th2 cells and CD103^+^ resident memory T cells (T_RM_), suggesting that the immune surveillance persists for years after treatment with 5-FU plus calcipotriol. Using a mouse DMBA-TPA tumorigenesis model, the authors confirmed that Th2 cells were necessary and sufficient for elimination of SCC-like skin lesions. Calcipotriol plus 5-FU was protective against these tumors only in the presence of CD4^+^ T cells. Protection was lost in IL-4 receptor-deficient mice and rescued by transfer of WT CD4^+^ T cells.

Calcipotriol application results in the production of thymic stromal lymphopoietin (TSLP), a potent inducer of Th2 responses, in keratinocytes of psoriasis lesions but not normal skin (15). Oka et al. demonstrated that 5-FU-plus–calcipotriol treatment specifically induced TSLP in keratinocytes of AK and SCC, but not in keratinocytes from unaffected skin, providing a mechanistic link between calcipotriol and the Th2 response (9). The authors also performed transcriptomic analysis of gene expression after 5-FU-plus–calcipotriol treatment and found that IL-24 was one of the most upregulated genes in treated AK (9). IL-24, also known as melanoma differentiation associated gene 7 (MDA7), has been reported to selectively induce cell death in a variety of solid tumor types (16). Human SCC cells, but not normal keratinocytes, were susceptible to IL-24–mediated cell death and autophagy in the presence of 5-FU. In addition, expression of IL-24 in SCC cells was induced by exposure to the canonical Th2 cytokine IL-4. Using mouse models of cSCC cancer induction, Oka and authors confirmed that type 2 immunity was critical for skin cancer surveillance. Constitutive expression of TSLP in mouse epidermis protected animals from SCC-like lesions, and expression of the TSLP receptor in CD4^+^ T cells was required for this protection. IL-24 was also required, as IL-24–deficient mice were not protected from SCC precursor development during calcipotriol-plus–5-FU treatment. Interestingly, calcipotriol-plus–5-FU treatment still induced marked Th2 cell infiltration to skin lesions but failed to prevent carcinogenesis in the absence of IL-24, underscoring that IL-24 is necessary for elimination of premalignant keratinocytes (9).

**Figure 1:**
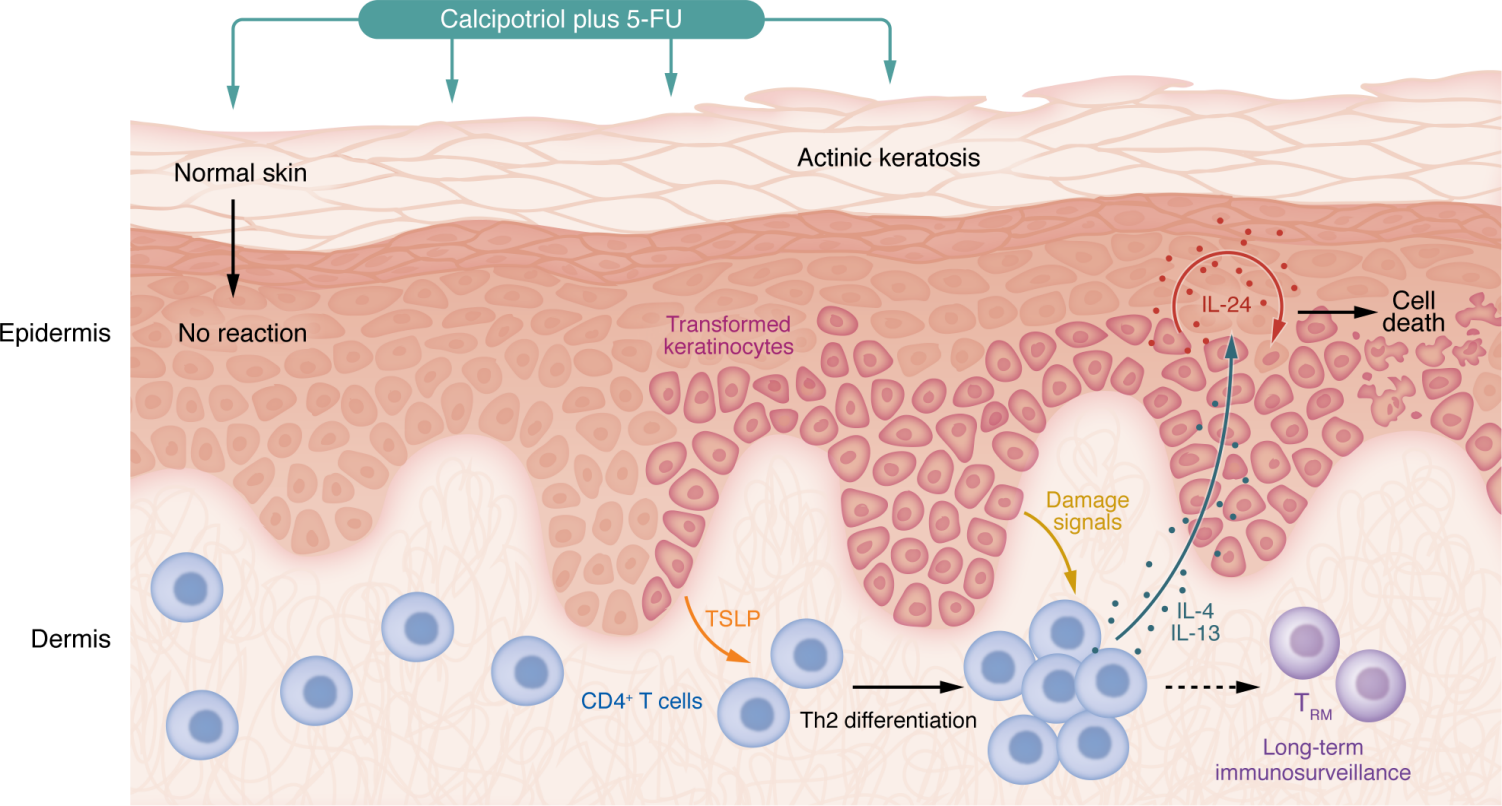
Topical calcipotriol plus 5-FU stimulates immune surveillance in actinic keratosis and squamous cell carcinoma. Sun-damaged skin harbors areas of normal epidermis mixed with areas of actinic keratosis, composed of transformed keratinocytes with dysregulated proliferation and differentiation, due to UV-induced somatic mutations. Topical calcipotriol induces TSLP production by transformed keratinocytes in actinic keratosis but not in unaffected keratinocytes. TSLP is recognized by T cells in the dermis and stimulates Th2 differentiation. Keratinocyte injury, mediated by the chemotherapeutic agent 5-FU, leads to expression of damage-associated molecular patterns including Annexin A1, Calreticulin, and MHC class II, which further promote T cell activation and proliferation. Activated Th2 cells secrete IL-4 and IL-13, which specifically stimulate transformed keratinocytes to express IL-24. IL-24 acts in an autocrine and paracrine fashion on transformed keratinocytes to enhance apoptosis, autophagy, and general cell death. In addition, ongoing stimulation of dermal T cells facilitates development of resident memory T cells (TRM) in the skin, allowing for long-term immunosurveillance and elimination of squamous cell carcinoma and its precursors.

Taken together, these findings provide a model (Figure 1) in which topical calcipotriol, in the context of cellular injury produced by 5-FU, induces TSLP expression selectively in AK keratinocytes. TSLP then induces differentiation and recruitment of Th2 cells to AK lesions in the skin, a process that is further enhanced by expression of damage signals such as Annexin A1, Calreticulin, and MHC class II in the epidermis. Th2 cells secrete IL-4 and IL-13, which specifically act on malignant or premalignant keratinocytes to induce IL-24 production. IL-24, acting via an autocrine or paracrine mechanism, then synergizes with 5-FU to enhance apoptosis, autophagy, and eliminate tumor cells (9).

Type 2 immunity has traditionally been viewed as favoring tumor growth. In contrast, cancer immunosurveillance and the elimination of cancer cells is viewed as predominately mediated by type 1 immunity, including Th1 cells, CD8^+^ T cells, and IFN-γ. However, recent evidence suggests that Th2 cells may either promote or fight against cancer based on the tissue context (17). For example, Th2 cells were found to promote pancreatic cancer growth (18), while adoptively transferred Th2 cells eliminated myeloma tumors (19). Similar conflicting results are also seen with IL-4 and TSLP. IL-4 signaling promotes protumorigenic myelopoiesis, driving non-small cell lung cancer, and blockade of IL-4R signaling enhanced cancer immunotherapy (20). In contrast, recombinant IL-4 potentiated type 1 immunity to eliminate cancer (21). TSLP has been found to either promote tumor destruction or tumor growth (22). Ultimately, the cancer subtype, tumor microenvironment, and immune contexture likely determine type 2 immunity’s role in cancer. Taken together, the effect of type 2 immunity on cancer is difficult to predict even in similar tissues. While Oka et al. (9) demonstrate that TSLP production from keratinocytes reduces cSCC formation, a recent study showed that keratinocyte-derived TSLP promoted melanoma growth and metastasis (23).

As with other type 2 cytokines, IL-24 has pleiotropic effects including anticancer properties and propagation of autoimmunity, including allergic diseases (24). Expression of IL-24 in chimeric antigen receptor T (CAR-T) cell therapy improved anticancer efficacy (25). In line with other reports, IL-24 and type 2 immunity, more broadly, are critical for barrier tissue (e.g., skin) function and repair (26). How IL-24 promotes keratinocyte proliferation in chronic inflammatory disease like atopic dermatitis and keratinocyte death in premalignant lesions remains to be determined.

## Clinical implications and unanswered questions

Analogous to the revolution in cancer treatment we have witnessed since the development of immune checkpoint inhibitors, Oka et al. (9) highlights the potential for harnessing immune-mediated tumor surveillance in cancer prevention. The authors demonstrate that the addition of calcipotriol to 5-FU field therapy results in development of CD103^+^ T_RM_s that persist for years, suggesting a durable prevention effect. It is likely that the combination of calcipotriol with 5-FU represents a substantial advance over existing field treatments for premalignant lesions, and it has already been reported to be effective for treatment of SCC in situ in a retrospective series (27). However, it is important to note that the clinical efficacy of calcipotriol plus 5-FU has not yet been demonstrated in a phase 3 clinical trial, and optimal dosing strategies have yet to be defined.

Several unanswered questions remain. If type 2 immunity is critical for immune surveillance of cancer in the skin, is it possible to promote Th2 signals in combination with systemic immune checkpoint inhibition to enhance treatment of advanced or metastatic cSCC? Are there specific neoantigens in AK and cSCC that are frequently targeted by tumor-specific Th2 cells? Because both immunosuppressed patients and patients with chronic skin inflammation exhibit markedly increased risk of cSCC development, can calcipotriol plus 5-FU effectively correct the immune dysfunction and promote tumor surveillance in these high-risk groups? We look forward to additional translational studies and clinical trials exploring skin cancer prevention via stimulated immune surveillance.

## Version 1 01/02/2025

Electronic publication
